# The inverted singlet–triplet gap: a vanishing myth?

**DOI:** 10.3389/fchem.2023.1239604

**Published:** 2023-07-27

**Authors:** Andreas Dreuw, Marvin Hoffmann

**Affiliations:** Interdisciplinary Center for Scientific Computing, Ruprecht-Karls University, Heidelberg, Germany

**Keywords:** singlet–triplet gap, excited states, quantum chemistry, electronic structure, exciton analysis

## Abstract

Molecules with an inverted singlet–triplet gap (STG) between the first excited singlet and triplet states, for example, heptazine, have recently been reported and gained substantial attention since they violate the famous Hund’s rule. Utilizing state-of-the-art high-level *ab initio* methods, the singlet–triplet gap vanishes and approaches zero from below whatever is improved in the theoretical description of the molecules: the basis set or the level of electron correlation. Seemingly, the phenomenon of inverted singlet–triplet gaps tends to vanish the closer we observe.

## 1 Introduction

The energy difference between the first excited singlet S_1_ and the corresponding first excited triplet T_1_ states is generally referred to as the singlet–triplet gap (STG). It makes sense to speak of the STG only between pairs of excited singlet and triplet states when they possess the same, or at least very similar, spatial electronic wave functions and differ essentially only in the spin part of the total wave functions. It is defined positive when the triplet state is lower than the singlet state, which is the natural order due to the favorable exchange interaction of the unpaired electrons, leading to an overall lower total energy of the triplet state than the singlet state. Previously, in 1927, Friedrich Hund postulated the energetic preference of an open-shell system toward its high-spin state, which became well-known in the field of chemistry as part of “Hund’s rules” (Hund, 1925, 1927).

The STG plays an important role in photochemistry as it is relevant for intersystem crossing (ISC) from the singlet to the triplet state manifold, which generally leads to a loss of fluorescence. A small STG, however, is also known to lead to thermally activated reverse intersystem crossing (RISC) from the triplet to the singlet state, indicated by subsequent fluorescence. This process is currently known as thermally activated delayed fluorescence (TADF), which is exploited for efficient organic light-emitting diodes (OLEDs) (Shi et al., 2022; Nakanotani et al., 2021).

Recently, several organic closed-shell molecules with exceptionally small singlet–triplet gaps between the first excited singlet S_1_ and triplet T_1_ states have been reported, with heptazines and cycl[3.3.3]azines among others holding great promise for their application as TADF emitters (Ehrmaier et al., 2019; De Silva, 2019; Ricci et al., 2021; Li et al., 2022) Interestingly, quantum-chemical calculations conducted on heptazine, cycl[3.3.3]azine, and related compounds revealed an inverted (negative) singlet–triplet gap, with the first excited singlet state possessing a lower total energy than the corresponding triplet state. These so-called inverted singlet–triplet (INVEST) molecules thus formally violate Hund’s rule.

Focusing on heptazine as the most widely theoretically studied INVEST molecule, the most reliable calculated STGs become all negative and singlet–triplet state inversion is observed. In Pollice et al. (2021), STGs of heptazine were reported ranging from −0.109 eV at the level of the third-order algebraic diagrammatic construction (ADC) scheme for excited states (ADC(3)) with a cc-pVDZ basis set up to −0.344 eV at the domain-based local pair natural orbital (DLNPO) n-electron valence perturbation theory of second order (NEVPT2) with six electrons in the six valence orbital (6,6) level of theory using the def2-SV(P) basis set. Equation-of-motion (EOM) coupled-cluster with singles and doubles (CCSD) yields −0.180 eV when canonical orbitals and the cc-pVDZ basis set are used, and −0.214 eV with frozen natural orbitals (FNOs). It has been noted that the linear-response time-dependent density functional theory (TDDFT) with standard exchange-correlation kernels yields consistently positive STGs for heptazine independent of the amount of non-local Hartree–Fock (HF) exchange (Bhattacharyya, 2021; Ricci et al., 2021; Tuckova et al., 2022). The larger the static amount of HF exchange in the xc-kernel, the higher positive the computed STG tends to be, which is not surprising since the STG is in first order driven by the exchange interaction of the unpaired electrons. In addition, long-range corrected functionals like *ω*B97XD yield an STG of 0.23 eV in combination with the Tamm–Dancoff approximation (TDA) (Ricci et al., 2021). Only when the spin-flip variant of TDDFT is applied or double-hybrid long-range-corrected xc-functionals are used, negative STGs are obtained (Pollice et al., 2021; Tuckova et al., 2022). At the DFT/MRCI level, STG of heptazine has been found to be close to zero, i.e., −0.01 eV (Dinkelbach et al., 2021), however, still conserving the negative sign. Based on these findings, the computational hunt for improved INVEST molecules for use in OLEDs started (Pios et al., 2021; Pollice et al., 2021; Sobolewski and Domcke, 2021; Sanz-Rodrigo et al., 2021; Li et al., 2022; Tuckova et al., 2022).

Recently, experimental evidence of small negative STGs in substituted derivatives of heptazine has been reported (Ehrmaier et al., 2019; Aizawa et al., 2022). Using time-resolved photoluminescence and transient absorption spectroscopy, the luminescence of a chemically stable heptazine derivative has been shown to be insensitive to the presence of external heavy atom sources and triplet-quenching oxygen, confirming its fluorescence characteristic and suggesting the lack of an ISC decay channel into a lower-lying triplet state (Ehrmaier et al., 2019). Temperature-dependent measurements of the fluorescence rate revealed the time constant of TADF to anomalously decrease with decreasing temperature, leading to the conclusion that the emissive singlet state is the lowest. Recent theoretical calculations of rate constants demonstrated the efficiency and temperature dependence of TADF do not exclusively depend on STG; moreover, the rates were determined by an interplay of vibrionic effects and near-zero STGs, i.e., practically degenerated S_1_ and T_1_ states, which were shown to be optimal for efficient RISC in OLEDs (Dinkelbach et al., 2021).

**FIGURE 1:**
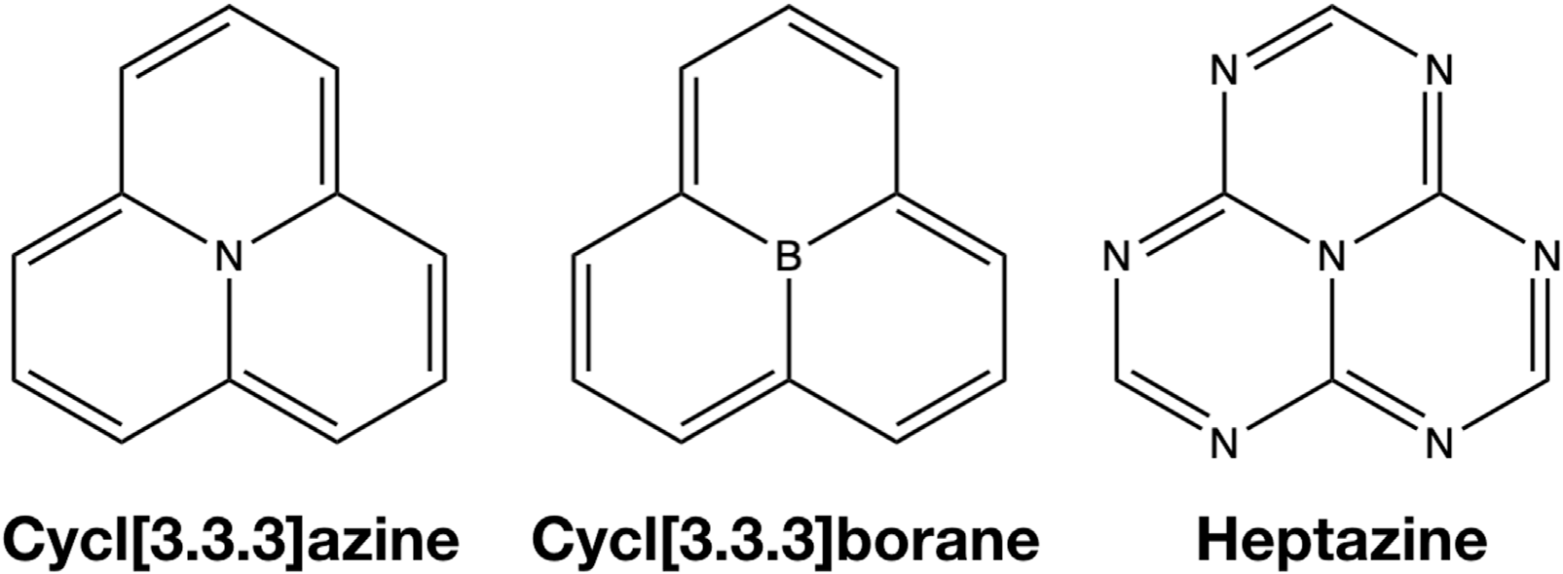
Lewis structures of cycl[3.3.3]azine, heptazine, and cycl[3.3.3]borane.

For a singlet–triplet state inversion to occur, the exchange interaction among the unpaired electrons needs to be small, and an additional mechanism stabilizing the singlet state over the triplet state needs to be present. Previously, this stabilization of the singlet state has been attributed to energetically low-lying doubly excited singlet configurations, with which the singlet state can interact but not the triplet state (De Silva, 2019). Within this work, we aim at gaining a deeper understanding of singlet–triplet state inversion and try to identify the physical mechanism leading to it. For this objective, we study the proto-typical INVEST molecules heptazine and cycl[3.3.3]azine, and also include the hypothetical boron derivative cycl[3.3.3]borane (Figure 1) in our studies to see the effect of switching from an electron-rich to an electron-deficient system on STG. High-level *ab initio* methods will be utilized including the effect of triples in the calculation of STGs as well. The electronic structures of the excited states will be analyzed using advanced analysis tools to obtain a better picture of their physical nature, possibly explaining the inversion of STG. We will see, however, that the only significant difference in the electronic structure of the singlet S_1_ and triplet T_1_ states, besides their spin states, is the entanglement between the particle and the hole in the corresponding singlet and triplet excitons. Moreover, the closer we look or, in other words, the more accurate we try to compute the STGs of heptazine, cycl[3.3.3]azine, or cycl[3.3.3]borane, the less negative they become, until all become positive at the highest affordable level of the electronic structural theory.

## 2 Computational details

All gas-phase geometry optimizations were performed at the RI-MP2/cc-pVTZ level of the theory using the PSI4 software package (Smith et al., 2020). The vertical excited states have been computed at the theoretical levels of the algebraic diagrammatic construction methods for the polarization propagator at the second and third orders of the perturbation theory (ADC(2) and ADC(3)) (Wormit et al., 2014; Schirmer, 1982; Harbach et al., 2014). STGs have also been computed as the difference in total energies of the first excited singlet S_1_ and triplet T_1_ states computed at the level of coupled cluster singles and doubles (CCSD), as well as CCSD with perturbative triples correction (CCSD(T)) (Purvis and Bartlett, 1982; Raghavachari et al., 1989), denoted in the following as *δ* values. For these calculations, the underlying unrestricted Hartree-Fock reference has been tweaked to converge with the open-shell singlet and triplet states, in which the highest occupied molecular orbital (HOMO) is replaced by the lowest unoccupied molecular orbital (LUMO) using the maximum overlap method (MOM) (Gilbert et al., 2008). The excited-state wave functions of S_1_ and T_1_ have been studied using exciton analyses, as implemented by the open-source software library for wavefunction analysis, libwfa (Plasser et al., 2022). For all excited-state calculations, the Q-Chem software package in version 5.2 was used (Epifanovsky et al., 2021).

## 3 Results

### 3.1 Excited state characteristics

**FIGURE 2:**
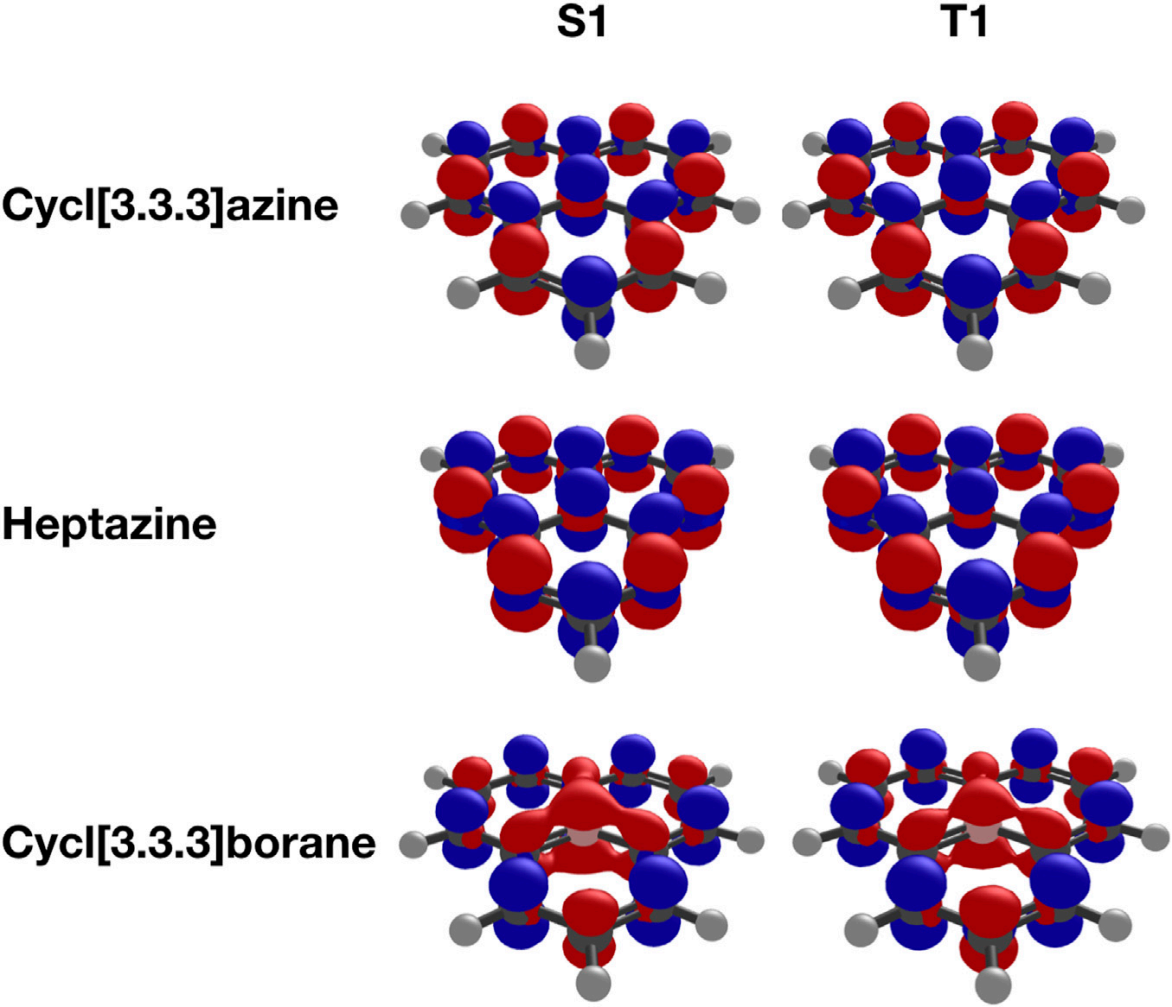
Difference densities (isovalue = 0.005) of cycl[3.3.3]azine, heptazine, and cycl[3.3.3]borane computed at the ADC(3)/cc-pVTZ level.

All three investigated molecules, namely, heptazine, cycl[3.3.3]azine, and cycl[3.3.3]borane, possess a symmetry-forbidden and thus spectroscopically dark excited singlet S_1_ state. This state is represented in the molecular orbital picture as essentially pure electronic transition of a single electron from the HOMO to the LUMO. The corresponding difference densities (Figure 2) reveal the expected ππ* characteristic of the S_1_ state in all three molecules. The corresponding first excited triplet T_1_ state exhibits an identical orbital composition, with one electron occupying the HOMO and another electron with identical spin occupying the LUMO of the ground-state configuration. A short observation at the difference densities of the S_1_ and T_1_ states in these molecules (Figure 2) reveals them to be indistinguishable from the singlet and triplet states in the same molecule. Although those between the nitrogen derivatives are practically identical, only the difference densities of cycl[3.3.3]borane are slightly different due to its electron-deficient nature compared to the nitrogen derivatives.

The vertical excitation energies of the S_1_ and T_1_ states at the ADC(3)/cc-pVTZ level are 0.81 and 0.87 eV for cycl[3.3.3]azine, 2.81 and 2.88 eV for heptazine, and 0.55 and 0.65 eV for cycl[3.3.3]borane. This results in negative STGs for all three molecules of −0.06, −0.07, and −0.10 eV at the equilibrium geometry of the singlet ground state S_0_. Seemingly, switching of an electron-rich system (azine) to an electron-deficient system (borane) leads to an increase in the negative singlet–triplet gap. The number of nitrogen atoms in heptazine compared to cycl[3.3.3]azine has, on the contrary, only a negligible influence. It is important to note that geometry optimization of the triplet state leads to only insignificant changes of the singlet–triplet gap, for example, from 0.063 to 0.067 eV in the case of cycl[3.3.3]azine at the ADC(3)/cc-pVTZ level. The induced geometric change upon excitation of a single electron in these systems is minimal due to the rigidity of the systems. The insignificant difference between vertical and adiabatic STGs of heptazine derivatives is further highlighted in the literature (Ehrmaier et al., 2019; Dinkelbach et al., 2021).

### 3.2 Excitonic properties of S_1_ and T_1_

Exciton analyses are insightful tools for complex electronic excited-state wave functions (Bäppler et al., 2014; Plasser and Lischka, 2012; Plasser et al., 2014b; Plasser et al., 2014b; Plasser et al., 2015; Mewes and Dreuw, 2019). They are based on the two-particle exciton wave function, which is obtained from the transition density matrix, and can be used to compute expectation values, thus studying the physical properties of the corresponding electronic state.

**FIGURE 3:**
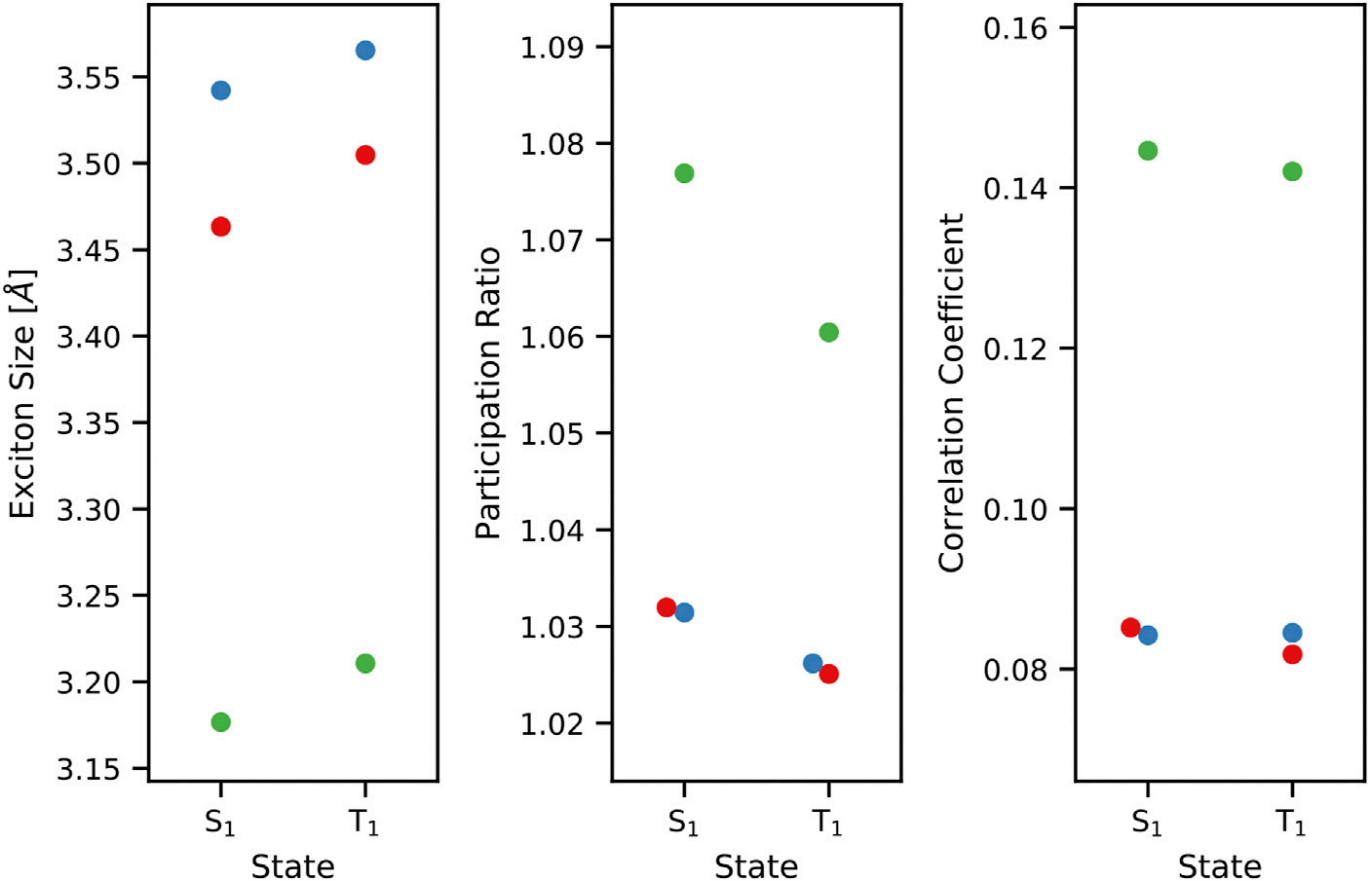
Exciton size (left), NTO participation ratio PR_*NTO*_ (middle), and the electron–hole correlation coefficient R_*eh*_ (right) of excitons corresponding to the S_1_ and T_1_ states of cycl[3.3.3]azine (blue), heptazine (green), and cycl[3.3.3]borane (red) at the theoretical level of ADC(3)/cc-pVTZ.

Following this general procedure, the spatial sizes of the singlet and triplet excitons of S_1_ and T_1_ of cycl[3.3.3]azine, heptazine, and cycl[3.3.3]borane have been computed. Interestingly, the excitons of cycl[3.3.3]azine and cycl[3.3.3]borane possess very similar sizes around 3.5 Å for both the S_1_ and T_1_ states. Heptazine, on the contrary, exhibits distinctively smaller singlet and triplet excitons of around 3.2 Å (Figure 3). The exact values are given in the Supplementary Material. However, the trend in the spatial extent of the excitons does not correlate with the trend in the observed STGs since heptazine and cycl[3.3.3]azine have similar STGs but differ in exciton sizes.

A second insightful exciton property is the spatial correlation of the hole and electron within the exciton. The correlation coefficient, defined as

$$R_{eh}=\frac{\left\langle r_{h}\cdot r_{e}\right\rangle -\left\langle r_{h}\right\rangle \cdot \left\langle r_{e}\right\rangle }{\sigma_{h}\sigma_{e}},$$ (1)

is positive when the electron and hole are spatially correlated, and negative when they are anti-correlated. *σ*_*h*_ and *σ*_*e*_ correspond to the root-mean-square sizes of the hole and electron distributions, respectively. As can be seen in Figure 3, the spatial electron–hole correlation of both states S_1_ and T_1_ increases from 0.08 in cycl[3.3.3]azine and cycl[3.3.3]borane to 0.14 in heptazine. Again, this trend in the computed correlation coefficients cannot be related to the calculated STGs.

An alternative measure to estimate the importance of electron correlation and the multi-configurational characteristic is to inspect the natural transition orbitals (NTOs) and their occupation numbers (Martin, 2003). In order to quantify how many pairs of NTOs are essential to characterize an electronic transition, the so-called natural transition orbital participation ratio (Luzanov and Zhikol, 2010; Plasser and Lischka, 2012) has been introduced:

$$PR_{NTO}=\frac{{\left(\sum_{i}\lambda_{i}\right)}^{2}}{\sum_{i}\lambda_{i}^{2}},$$ (2)

with *λ*_*i*_ representing the weights of the respective NTO pair configurations. Inspection of the values of PR_*NTO*_ for the S_1_ and T_1_ states of cycl[3.3.3]azine, heptazine, and cycl[3.3.3]borane reveals them to be described by a single NTO pair. They exhibit a negligible multi-configurational characteristic with values for PR_*NTO*_ close to one. The only possibly significant difference that can be seen for the singlet and triplet states of heptazine is where PR_*NTO*_ is slightly larger for the singlet state than for the triplet state (Figure 3).

To corroborate this initial finding, we analyze the excited singlet and triplet states further by computing the entanglement entropy between the electron and hole according to

$$\begin{array}{cc} S_{H|E}= & -tr\left[{\hat{\rho }}_{E}\log_{2}{\hat{\rho }}_{E}\right]\\ = & tr\left[{\hat{\rho }}_{H}\log_{2}{\hat{\rho }}_{H}\right]\\ = & \underset{i}{\sum }\lambda_{i}\log_{2}\lambda_{i}, \end{array}$$ (3)

as well as the corresponding number of entangled states via

$$Z_{H|E}=2^{S_{H|E}}=1/\underset{i}{\prod }\lambda_{i}^{\lambda_{i}},$$ (4)

where ${\hat{\rho }}_{E}$ and ${\hat{\rho }}_{H}$ are the density operators defined for the electron and hole subsystems, for which NTOs are the eigenfunctions (Plasser, 2016). Subsequently, the transition density, i.e., the electron and hole subsystems can be analyzed with respect to their von Neumann entanglement entropy (Bester et al., 2004). In principle, the entanglement entropy quantifies the correlation between the electron and hole, and it can, thus, serve as a criterion for how well excited states are described at a certain level of the theory, or in other words, how converged the theoretical description is when going from a lower to a higher level of the theory. In the case of well-described states, the entanglement entropy should not change when higher levels of the theory are employed.

At the ADC(2) level, S_*H*|*E*_ has values of 0.115 and 0.091 for S_1_ and T_1_ of cycl[3.3.3]azine, respectively, revealing the electron and the hole to be strongly correlated in the triplet state than that in the singlet state. Going with ADC(3), the entanglement entropy increases to 0.239 and 0.135 with a substantially larger value for the singlet state than for the triplet state, while the number of entangled states Z_*H*|*E*_ does not change and instead supports the finding of PR_*NTO*_. Seemingly, the description of the excitons is not yet converged at the level of ADC(2), in particular in one of the singlet state, since S_*H*|*E*_ changes strongly. Going with heptazine, the hole and electron are overall strongly entangled than in cycl[3.3.3]azine that are already at the ADC(2) levels with values of 0.160 and 0.139 for S_1_ and T_1_, respectively. Furthermore, going with ADC(3), the entanglement entropy increases substantially to 0.299 and 0.242 for S_1_ and T_1_, respectively, while Z_*H*|*E*_ remains unchanged, yet demonstrating that at least a third-order treatment of the states in question is required for their proper theoretical description. Although at the ADC(2) level, the singly excited states are described consistently in the second order of the perturbation theory similar to the electronic ground state in the MP2 theory, at the ADC(3) level, their description is at the third order of the perturbation theory.

Surprisingly, the inverted STG of cycl[3.3.3]azine, heptazine, and cycl[3.3.3]borane becomes smaller when going from ADC(2) to ADC(3), for example, from −0.24 eV to only −0.07 eV in the case of heptazine. This contrasts the general notion that an improved description of electron correlation stabilizes the singlet state over the triplet state, and being the origin of the occurrence of singlet–triplet state inversion. The question, thus, arises what effect an improved description of electron correlation within the employed *ab initio* excited state method has on the computed STG.

### 3.3 Influence of the electron correlation

In previous works, the occurrence of inverted STGs has been related to doubly excited configurations in the electronic singlet wave functions, stabilizing the singlet state over the triplet state (De Silva, 2019). Doubly excited configurations can have, however, different meanings: i) they are present in correlated ground states and can occur in excited states, when the correlation pattern changes; ii) they can refer to genuinely doubly excited states, and iii) they are also related to orbital relaxation effects in singly excited states.

The analysis of the contributing molecular orbitals, including NTOs, PR_*NTO*_, and Z_*H*|*E*_, all revealed the S_1_ and T_1_ states of cycl[3.3.3]azine, heptazine, and cycl[3.3.3]borane to essentially correspond to single-electron transitions from HOMO to LUMO. For the investigation of the influence of doubly excited configurations on these genuinely singly excited states, the ADC methods for excited states provide an ideal testing ground since their influence is increased in a well-defined manner going from ADC(2) to ADC(3) via the intermediate extended ADC(2) (ADC(2)-x) scheme. ADC(2) contains doubly excited states explicitly only at the zeroth order and the coupling between doubly and singly excited states at the first order of the perturbation theory. However, dynamic correlation effects are nevertheless included and contained in the matrix elements of the singles' block of the ADC(2) matrix via the second-order perturbation theory similar to MP2 and closely related to configuration interaction singles with perturbative doubles (CIS(D)) (Head-Gordon et al., 1994). A particularly useful diagnostics for the influence of explicit doubly excited configurations is ADC(2)-x, in which matrix elements of the doubles block are increased to the first order, while the coupling between singles and doubles remains in ADC(2) at the first order (Trofimov and Schirmer, 1995; Starcke et al., 2006). Thus, essentially, only the explicit influence of the doubles on the singles is increased, while the dynamic correlation in the singles block remains the same. At the ADC(3) level, the singles block and the coupling block between singles and doubles are finally increased up to the third and second orders in the perturbation theory, resulting in a consistent third-order description of the single excitations; here, the S_1_ and T_1_ states of cycl[3.3.3]azine, heptazine, and cycl[3.3.3]borane are provided.

**FIGURE 4:**
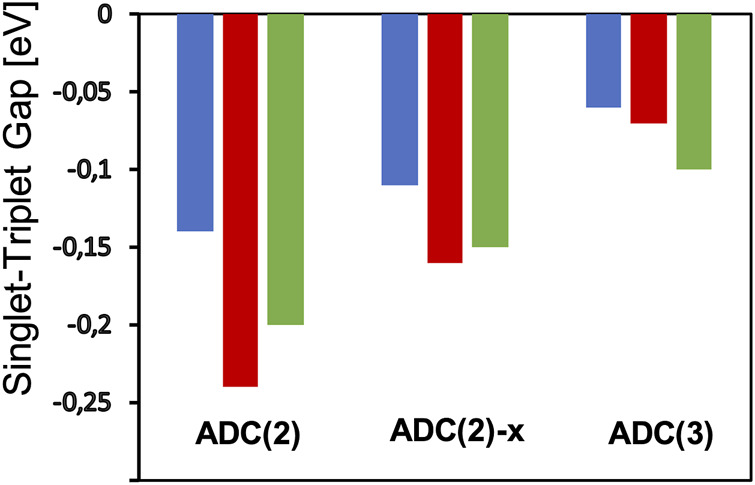
Calculated singlet–triplet gaps of cycl[3.3.3]azine (blue), heptazine (green), and cycl[3.3.3]borane (red) at the theoretical levels of ADC(2), ADC(2)-x, and ADC(3) in combination with the cc-pVTZ basis set.

At the ADC(2) level, the STGs of cycl[3.3.3]azine, heptazine, and cycl[3.3.3]borane have values of −0.14, −0.24, and −0.20 eV, respectively, showing the expected singlet–triplet state inversion (see Figure 4; Supplementary Table S1). Although STGs are positive throughout ADC(1), the influence of dynamic correlation on the singlet and triplet states, as included in the matrix elements of the singles block of the ADC(2) matrix, leads to the stabilization of S_1_ over T_1_ and are indeed needed to obtain a negative STG (De Silva, 2019). Going with ADC(2)-x and thereby increasing the explicit influence of the doubly excited configurations lead to STGs of −0.11, −0.16, and −0.15 eV for these three molecules, i.e., the negative STG becomes consistently smaller (Figure 4). The inclusion of doubly excited configurations at the ADC level, thus, stabilizes the triplet T_1_ state again slightly over the singlet S_1_ state. Going further with ADC(3), the negative STGs become even smaller with values of −0.06, −0.07, and −0.10 eV when the description of the singles themselves is increased up to the third order of the perturbation theory, including higher-order correlation effects (Figure 4). This has also manifested itself in the increase of the electron–hole entanglement S_*H*|*E*_ within these states when going from ADC(2) to ADC(3), which was more pronounced in S_1_ than T_1_.

A second, alternative pathway to include higher-order correlation effects into the calculation of STGs is to resort to high-level ground-state correlation methods and to tweak the reference wave function to converge into the lowest excited triplet state and to the first excited singlet state. The difference in the obtained total energies then corresponds with STG. For this objective, we use the established CCSD and CCSD(T) methods in connection with a correlation-consistent triple-zeta basis set, which is mandatory to capture the electron correlation. In particular, ΔCCSD(T) was recently demonstrated to yield an excellent description of excited states with resulting excitation energies rivaling EOM-CCSDT in accuracy (Lee et al., 2019). We thus expect the values obtained for the STG at the CCSD(T) level to be the most reliable values in our investigation.

**FIGURE 5:**
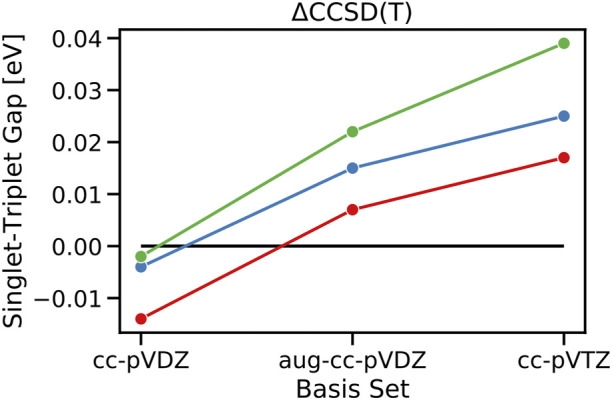
Calculated singlet–triplet gaps of cycl[3.3.3]azine (blue), heptazine (green), and cycl[3.3.3]borane (red) at the theoretical level of ΔCCSD(T) in combination with basis sets of increasing quality from cc-pVDZ, aug-cc-pVDZ, and cc-pVTZ.

First, inspecting the values of STGs at the CCSD level, they are completely negative with values of −0.303, −0.382, and −0.364 at the ΔCCSD level for cycl[3.3.3]azine, heptazine, and cycl[3.3.3]borane when the cc-pVTZ basis set is used. This is in agreement with the previous findings in the literature and our previous observation that dynamic correlation via the inclusion of doubles stabilizes the singlet state over the triplet state. Moving on to ΔCCSD(T) in combination with the cc-pVTZ basis, however, the STGs of all three investigated molecules change signs and become 0.025, 0.039, and 0.017 eV for cycl[3.3.3]azine, heptazine, and cycl[3.3.3]borane, respectively (Figure 5). In agreement with the previous observation at different ADC levels of the theory, where the increase from the second to third order of the perturbation theory has led to a decrease in the inverted STGs, the inclusion of triples in the CCSD(T) calculation reveals the same trend. This shows that triples are needed to capture higher-order correlation effects present in the S_1_ and T_1_ states, which are related to the entanglement of the hole and particle in the corresponding exciton, as previously analyzed. At the ΔCCSD(T) level, the energy of the triplet T_1_ state is lowered so much over that of the singlet S_1_ state that the previously inverted STGs turn into regular STGs again, obeying Hund’s rule.

The employed UHF open-shell singlet reference employed in our ΔCCSD and ΔCCSD(T) calculations is not a spin-pure state. However, we obtain large negative STGs for ΔCCSD and positive gaps for ΔCCSD(T), and both use the same underlying CCSD wave function with the same spin contamination. The S^2^ operator exhibits expectation values of 1.042, 1.034, and 1.045 for the open-shell singlet state of cycl[3.3.3]azine, heptazine, and cycl[3.3.3]borane and 2.014, 2.014, and 2.012 for the corresponding T_1_ states at the CCSD level, respectively. Although the triplet state does not suffer from spin contamination, the open-shell singlet ground state represents a mixture of S_1_ and T_1_, and due to the linearity of the quantum mechanics, its energy should lie between the singlet and the triplet states. Consequently, the possible error introduced by singlet–triplet state mixing, i.e., spin contamination, is expected to lead to an underestimation of the absolute values of the calculated gaps. The sign switch of STGs is thus due to the inclusion of triply excited configurations only, as already indicated in our exciton analyses at ADC(2) and ADC(3) levels. Therefore, it does not hamper the qualitative conclusion drawn that the negative STGs seem to vanish the more accurately we look.

**FIGURE 6:**
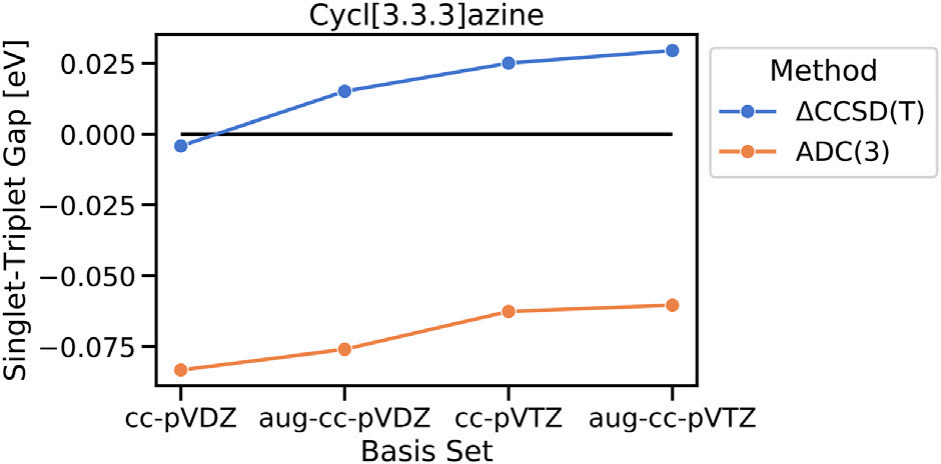
Basis-set dependence of the calculated singlet–triplet gap of cycl[3.3.3]azine on ΔCCSD(T) and ADC(3) levels of the theory.

The importance of higher-order electron correlation for an accurate description of STGs is further seen in their basis set dependence at ΔCCSD(T) and ADC(3) levels. Increasing the basis set size from cc-pVDZ to aug-cc-pVDZ leads to all three cases already switching to the sign of STGs with values of 0.015 eV, 0.022 eV, and 0.07 eV for cycl[3.3.3]azine, heptazine, and cycl[3.3.3]borane, respectively, and further increases to 0.017 eV, 0.025 eV, and 0.039 eV when going with cc-pVTZ. In order to confirm the observed trend of an increasing gap with the increasing basis set size, a single computationally very demanding ΔCCSD(T)/aug-cc-pVTZ calculation has been performed for cycl[3.3.3]azine, resulting in an even further increased STG of 0.029 eV (Figure 6). The same trend is also observed at the ADC(3) level, at which the inverted STG of cycl[3.3.3]azine becomes continuously smaller when the basis set size is increased. This finding has been noted earlier, i.e., extended basis sets are needed for the reliable computation of the STG of methylene (Carter and Goddard, 1987).

## 4 Conclusion and outlook

Setting out to understand the origin and the physical reasons for the inverted STG in heptazine derivatives and related triangular compounds with the help of high-level electronic structure methods and advanced excited-state wave function analysis tools, we noted that all inverted STGs will vanish the more we improved the applied electronic structure theory. At the highest applied level of the theory, the originally negative STGs eventually turned positive, raising a huge question regarding the existence of singlet–triplet state inversion in general. Based on our analysis, the entanglement or, in the quantum chemical language, the correlation between the electron and hole is not sufficiently accurately captured when only second-order methods considering only doubly-excited configurations are used. These higher-order correlations require at least the third-order methods including the effect of triples, as impressively shown in our results at ADC(3) and ΔCCSD(T) levels.

We are aware that these theoretical findings are in contrast when compared to the recently reported experimentally derived conclusions (Ehrmaier et al., 2019; Aizawa et al., 2022). First, the experiments were performed on substituted derivatives of heptazine, which are presently too large to compute their STGs directly using CCSD(T)/cc-pVTZ; however, the reported theoretical results at lower levels of the theory (−0.012 eV at the EOM-CCSD level (Aizawa et al., 2022)) for these derivatives are such that a re-inversion of STG to a regular STG does not appear unlikely. Second, the experiments are performed in solution whose influence is not considered in this work. Our initial preliminary studies including explicit solvent molecules and a polarizable continuum model, however, indicate a further small decrease in the inverted STGs rather than an increase. The influence on S_1_ and T_1_ is also seen to be very similar due to the very closely related electronic structure of these states such that an environment will most likely not have a large differential effect. This will be investigated in detail in the future. Third, the observation of an inverted temperature dependence of TADF signals does not necessarily mean that the corresponding singlet and triplet states are also inverted; one could, for example, also freeze out an alternative non-radiative fluorescence quenching channel, which dominates at higher temperatures. As noted previously, the efficiency of TADF depends on the details of the dynamical processes and not solely on the STG (Dinkelbach et al., 2021). In particular, it has been shown that the inclusion of zero-point vibrational energy corrections shifts the STG from −0.01 eV to 0.07 eV at the employed DFT/MRCI level of theory (Dinkelbach et al., 2021). Last, but not least, the molecular system sizes of heptazine derivatives are very large for the quantum chemical methods required to quantitatively predict the STGs, or in other words, to reach chemical accuracy. It is thus not certain what sign of the STG even higher levels of electronic structure would predict. The STGs of heptazine and related compounds are small, very small, and their accurate determination including their sign remains, thus, a challenge for both theory and experiments.

## Data Availability

The original contributions presented in the study are included in the article/Supplementary Material; further inquiries can be directed to the corresponding author.

## References

[B1] Aizawa N. Pu Y.-J. Harabuchi Y. Nihonyanagi A. Ibuka R. Inuzuka H. (2022). Delayed fluorescence from inverted singlet and triplet excited states. Nature 609, 502–506. 10.1038/s41586-022-05132-y 36104553 PMC9477729

[B2] Bäppler S. A. Plasser F. Wormit M. Dreuw A. (2014). Exciton analysis of many-body wavefunctions: Bridging the gap between the quasi-particle and molecular orbital pictures. Phys. Rev. A 90, 052521. 10.1103/PhysRevA.90.052521

[B3] Bester G. Shumway J. Zunger A. (2004). Theory of excitonic spectra and entanglement engineering in dot molecules. Phys. Rev. Lett. 93, 047401. 10.1103/PhysRevLett.93.047401 15323791

[B4] Bhattacharyya K. (2021). Can tddft render the electronic excited states ordering of azine derivative? A closer investigation with dlpno-steom-ccsd. Chem. Phys. Lett. 779, 138827. 10.1016/j.cplett.2021.138827

[B5] Carter E. C. Goddard W. A. III (1987). Electron correlation, basis sets, and the methylene singlet–triplet gap. J. Chem. Phys. 86, 862–865. 10.1063/1.452287

[B6] De Silva P. (2019). Inverted singlet-triplet gaps and their relevance to thermally activated delayed fluorescence. J. Phys. Chem. Lett. 10, 5674–5679. 10.1021/acs.jpclett.9b02333 31483656

[B7] Dinkelbach F. Bracker M. Kleinschmidt M. Marian C. M. (2021). Large inverted singlettriplet energy gaps are not always favorable for triplet harvesting: Vibronic coupling drives the (reverse) intersystem crossing in heptazine derivatives. J. Phys. Chem. A 125, 10044–10051. 10.1021/acs.jpca.1c09150 34756038

[B8] Ehrmaier J. Rabe E. J. Pristash S. R. Corp K. L. Schlenker C. W. Sobolewski A. L. (2019). Singlet-triplet inversion in heptazine and in polymeric carbon nitrides. J. Phys. Chem. A 123, 8099–8108. 10.1021/acs.jpca.9b06215 31466450

[B9] Epifanovsky E. Gilbert A. T. B. Feng X. Lee J. Mao Y. Mardirossian N. (2021). Software for the frontiers of quantum chemistry: An overview of developments in the q-chem 5 package. J. Chem. Phys. 155, 084801. 10.1063/5.0055522 34470363 PMC9984241

[B10] Gilbert A. T. Besley N. A. Gill P. M. (2008). Self-consistent field calculations of excited states using the maximum overlap method (mom). J. Phys. Chem. A 112, 13164–13171. 10.1021/jp801738f 18729344

[B11] Harbach P. H. P. Wormit M. Dreuw A. (2014). The third-order algebraic diagrammatic construction method (adc(3)) for the polarization propagator for closed-shell molecules: Efficient implementation and benchmarking. J. Chem. Phys. 141, 064113. 10.1063/1.4892418 25134557

[B12] Head-Gordon M. Rico R. J. Oumi M. Lee T. J. (1994). A doubles correction to electronic excited states from configuration interaction in the space of single substitutions. Chem. Phys. Lett. 219, 21–29. 10.1016/0009-2614(94)00070-0

[B13] Hund F. (1925). Large inverted singlet–triplet energy gaps are not always favorable for triplet harvesting: Vibronic coupling drives the (reverse) intersystem crossing in heptazine derivatives. Z. Phys. 33, 345. 10.1021/acs.jpca.1c09150 34756038

[B14] Hund F. (1927). Linienspektren und periodisches System der Elemente. Berlin: Springer. 10.1007/978-3-7091-5695-7

[B15] Lee J. Small D. W. Head-Gordon M. (2019). Excited states via coupled cluster theory without equation-of-motion methods: Seeking higher roots with application to doubly excited states and double core hole states. J. Chem. Phys. 151, 214103. 10.1063/1.5128795 31822103

[B16] Li J. Li Z. Liu H. Gong H. Zhang J. Yao Y. (2022). Organic molecules with inverted singlet-triplet gaps. Front. Chem. 10, 999856. 10.3389/fchem.2022.999856 36092667 PMC9448862

[B17] Luzanov A. V. Zhikol O. A. (2010). Electron invariants and excited state structural analysis for electronic transitions within cis, rpa, and tddft models. Int. J. Quant. Chem. 110, 902–924. 10.1002/qua.22041

[B18] Martin R. L. (2003). Natural transition orbitals. J. Chem. Phys. 118, 4775–4777. 10.1063/1.1558471

[B19] Mewes S. A. Dreuw A. (2019). Density-based descriptors and exciton analyses for visualizing and understanding the electronic structure of excited states. Phys. Chem. Chem. Phys. 21, 2843–2856. 10.1039/C8CP07191H 30687866

[B20] Nakanotani H. Tsuchiya Y. Adachi C. (2021). Thermally-activated delayed fluorescence for light-emitting devices. Chem. Lett. 50, 938–948. 10.1246/cl.200915

[B21] Pios S. Huang X. Sobolewski A. L. Domcke W. (2021). Triangular boron carbon nitrides: An unexplored family of chromophores with unique properties for photocatalysis and optoelectronics. Chem. Phys. Phys. Chem. 23, 12968–12975. 10.1039/d1cp02026a 34059871

[B22] Plasser F. Bäppler S. Wormit M. Dreuw A. (2014a). New tools for the systematic analysis and visualization of electronic excitations. II. Applications. J. Chem. Phys. 141, 024107. 10.1063/1.4885820 25027999

[B23] Plasser F. (2016). Entanglement entropy of electronic excitations. J. Chem. Phys. 144, 194107. 10.1063/1.4949535 27208936

[B24] Plasser F. Lischka H. (2012). Analysis of excitonic and charge transfer interactions from quantum chemical calculations. J. Chem. Theory Comput. 8, 2777–2789. 10.1021/ct300307c 26592119

[B25] Plasser F. Lrylov A. Dreuw A. (2022). libwfa: Wavefunction analysis tools for excited and open-shell electronic states. WIRES Comput. Mol. Sci. 12, e1595. 10.1002/wcms.1595

[B26] Plasser F. Thomitzni B. Bäppler S. A. Wenzel J. Rehn D. R. Wormit M. (2015). Statistical analysis of electronic excitation processes: Spatial location, compactness, charge transfer, and electron-hole correlation. J. Comp. Chem. 36, 1609–1620. 10.1002/jcc.23975 26119286

[B27] Plasser F. Wormit M. Dreuw A. (2014b). New tools for the systematic analysis and visualization of electronic excitations. Part i: Formalism. J. Chem. Phys. 141, 024106. 10.1063/1.4885819 25027998

[B28] Pollice R. Friederich P. Lavigne C. dos Passos Gomes G. Aspuru-Guzik A. (2021). Organic molecules with inverted gaps between first excited singlet and triplet states and appreciable fluorescence rates. Matter 4, 1654–1682. 10.1016/j.matt.2021.02.017

[B29] Purvis G. D. III Bartlett R. J. (1982). A full coupled-cluster singles and doubles model: The inclusion of disconnected triples. J. Chem. Phys. 76, 1910–1918. 10.1063/1.443164

[B30] Raghavachari K. Trucks G. W. Pople J. A. Head-Gordon M. (1989). A fifth-order perturbation comparison of electron correlation theories. Chem. Phys. Lett. 157, 479–483. 10.1016/S0009-2614(89)87395-6

[B31] Ricci G. San-Fabián E. Olivier Y. Sancho-García J. C. (2021). Singlet-triplet excited-state inversion in heptazine and related molecules: Assessment of td-dft and *ab initio* methods. ChemPhysChem 22, 553–560. 10.1002/cphc.202000926 33325598

[B32] Sanz-Rodrigo J. Ricci G. Olivier Y. Sancho-García J. C. (2021). Negative singlet–triplet excitation energy gap in triangle-shaped molecular emitters for efficient triplet harvesting. J. Phys. Chem. A 125, 513–522. 10.1021/acs.jpca.0c08029 33401898

[B33] Schirmer J. (1982). Beyond the random-phase approximation: A new approximation scheme for the polarization propagator. Phys. Rev. A 26, 2395–2416. 10.1103/PhysRevA.26.2395

[B34] Shi Y.-Z. Wu H. Wang K. Yu J. Ou X.-M. Zhang X.-H. (2022). Recent progress in thermally activated delayed fluorescence emitters for nondoped organic light-emitting diodes. Chem. Sci. 13, 3625–3651. 10.1039/D1SC07180G 35432901 PMC8966661

[B35] Smith D. G. A. Burns L. A. Simmonett A. C. Parrish R. M. Schieber M. C. Galvelis R. (2020). Psi4 1.4: Open-source software for high-throughput quantum chemistry. J. Chem. Phys. 152, 184108. 10.1063/5.0006002 32414239 PMC7228781

[B36] Sobolewski A. L. Domcke W. (2021). Organic molecules with inverted gaps between first excited singlet and triplet states and appreciable fluorescence rates. J. Phys. Chem. Lett. 12, 6852–6860. 10.1021/acs.jpclett.1c01926 34279950

[B37] Starcke J. H. Wormit M. Schirmer J. Dreuw A. (2006). How much double excitation character do the lowest excited states of linear polyenes have? Chem. Phys. 329, 39–49. 10.1016/j.chemphys.2006.07.020

[B38] Trofimov A. B. Schirmer J. (1995). An efficient polarization propagator approach to valence electron excitation spectra. J. Phys. B 28, 2299–2324. 10.1088/0953-4075/28/12/003

[B39] Tuckova L. Straka M. Valiev R. R. Sundholm D. (2022). On the origin of the inverted singlet–triplet gap of the 5th generation light-emitting molecules. Phys. Chem. Chem. Phys. 24, 18713–18721. 10.1039/d2cp02364d 35899835

[B40] Wormit M. Rehn D. R. Harbach P. H. Wenzel J. Krauter C. M. Epifanovsky E. (2014). Investigating excited electronic states using the algebraic diagrammatic construction (ADC) approach of the polarisation propagator. Mol. Phys. 112, 774–784. 10.1080/00268976.2013.859313

