# Supplementary material for: The inverted singlet–triplet gap: a vanishing myth?
Source: Front Chem. 2023 Jul 27;11:1239604. doi: 10.3389/fchem.2023.1239604 (PMC10413108; doi:10.3389/fchem.2023.1239604)
Supplement: Supplementary file 1 [file DataSheet1.PDF]

Supplementary Material

1 VERTICAL EXCITATION ENERGIES

Method	$\omega(S_1)$	$\omega(T_1)$	STG
Cycl[3.3.3]azine			
ADC(2)	1.02	1.61	-0.14
ADC(2)-x	0.32	0.43	-0.11
ADC(3)	0.81	0.87	-0.06
Heptazine			
ADC(2)	2.68	2.92	-0.24
ADC(2)-x	2.01	2.17	-0.16
ADC(3)	2.81	2.88	-0.07
Cycl[3.3.3]borane			
ADC(2)	0.78	0.98	-0.20
ADC(2)-x	0.08	0.23	-0.15
ADC(3)	0.55	0.65	-0.10

Table S1. Excitation energies ω of the first excited singlet S_1 abd triplet T_1 states of cycl[3.3.3]azine, heptazine and Cycl[3.3.3]borane as well as the corresponding singlet-triplet energy gap calculated at the theoretical levels of ADC(2), ADC(2)-x and ADC(3) using the cc-pVTZ basis set. All energies are given in eV.

2 EXCITED STATE PROPERTIES USING EXCITON ANALYSES

	Cycl[3.3.3]azine				*Heptazine*			
	ADC(2)		ADC(3)		ADC(2)		ADC(3)	
	S_1	T_1	S_1	T_1	S_1	T_1	S_1	T_1
r(exc) [Å]	3.54	3.57	3.53	3.55	3.19	3.27	3.18	3.21
d(e-h) [Å]	0.00	0.00	0.00	0.00	0.00	0.00	0.00	0.00
R(exc)	0.069	0.063	0.086	0.087	0.125	0.097	0.145	0.142
S(exc)	0.115	0.091	0.160	0.139	0.239	0.135	0.299	0.242
Z(exc)	1.083	1.065	1.117	1.101	1.180	1.098	1.230	1.183
PR(NTO)	1.021	1.016	1.032	1.027	1.057	1.028	1.077	1.060

Table S2. Results of excited state analyses of the lowest singlet S_1 and triplet T_1 states of Cycl[3.3.3]azine and Heptazine at ADC(2) and ADC(3) level using the cc-pVTZ basis set. The size of the corresponding exciton r(exc) and the electron-hole distance d(e-h) are given in Å. The correlation coefficient of the exciton R(exc), the entanglement entropy of the exciton, the number of entangled states Z(exc) and the participation ration of different natural transition orbitals PR(NTO) are given as unitless numbers.

3 ΔCCSD AND ΔCCSD(T) RESULTS

Method	$E(S_1)$ [a.u.]	$E(T_1)$ [a.u.]	STG [eV]
	Cycl[3.3.3]azine		
CCSD/cc-pVDZ	-515.84091774	-515.82972704	-0.305
CCSD/aug-cc-pVDZ	-515.91570680	-515.90527039	-0.284
CCSD/cc-pVTZ	-516.30101219	-516.28986027	-0.303
CCSD(T)/cc-pVDZ	-515.91717623	-515.91702184	-0.004
CCSD(T)/aug-cc-pVDZ	-516.00173197	-516.00228599	0.015
CCSD(T)/cc-pVTZ	-516.41231955	-516.41323849	0.025
	Heptazine		
CCSD/cc-pVDZ	-611.91248919	-611.89857701	-0.379
CCSD/aug-cc-pVDZ	-612.01462005	-612.00118171	-0.366
CCSD/cc-pVTZ	-612.44458279	-612.43054687	-0.382
CCSD(T)/cc-pVDZ	-611.99529414	-611.99522279	-0.002
CCSD(T)/aug-cc-pVDZ	-612.1092571	-612.11007012	0.022
CCSD(T)/cc-pVTZ	-612.56718734	-612.56863029	0.039
	Cycl[3.3.3]borane		
CCSD/cc-pVDZ	-486.01289325	-485.99946436	-0.365
CCSD/aug-cc-pVDZ	-486.07649233	-486.06387221	-0.343
CCSD/cc-pVTZ	-486.43636789	-486.42300102	-0.364
CCSD(T)/cc-pVDZ	-486.08631524	-486.08578419	-0.014
CCSD(T)/aug-cc-pVDZ	-486.15823675	-486.15848707	0.007
CCSD(T)/cc-pVTZ	-486.54202154	-486.54263513	0.017

Table S3. Total energies of the S_1 and T_1 states of Cycl[3.3.3]azine, Heptazine and Cycl[3.3.3]borane calculated at the couples cluster singles and doubles (CCSD) and CCSD plus perturbative triples correction CCSD[T] level of theory given in atomic units (hartrees) as well as the resulting triplet gap (STG) given in eV.

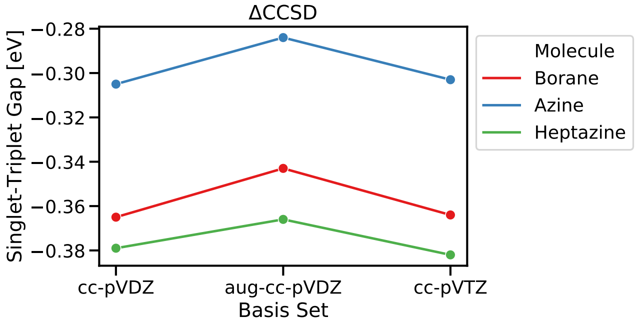

Figure S1. Calculated singlet-triplet gaps of Cycl[3.3.3]azine, Heptazine and Cycl[3.3.3]borane at ΔCCSD level using basis sets of improving quality from cc-pVDZ, aug-cc-pVDZ to cc-pVTZ basis sets.
